# Immigrant workers in the meat industry during COVID-19: comparing governmental protection in Germany, the Netherlands, and the USA

**DOI:** 10.1186/s12992-025-01104-9

**Published:** 2025-03-22

**Authors:** Nora Gottlieb, Ingrid Jungwirth, Marius Glassner, Tesseltje de Lange, Sandra Mantu, Linda Forst

**Affiliations:** 1 Department of Population Medicine and Health Services Research (AG2), School of Public Health, Bielefeld University, P. O. Box 10 01 31, D-33501 Bielefeld, Germany; 2 Faculty of Society and Economics, Rhine-Waal University of Applied Sciences, Marie-Curie-Str. 1, 47533 Kleve, Germany; 3 Faculty of Law, Centre for Migration Law, Radboud University, P.O. Box 9049, 6500 KK Nijmegen, The Netherlands; 4 Environmental and Occupational Health Sciences, School of Public Health, University of Illinois, 1603 W. Taylor Street, 60612 Chicago, IL USA

**Keywords:** COVID-19, Emergency preparedness, Food systems, Immigrant labor, Labor migration, Meat industry, Occupational safety and health, Policy analysis, Precarious work

## Abstract

The meat industry showcases the precarity of employment arrangements as part of broader global economic liberalization. In many countries, its workforce consists mostly of precariously employed immigrant and resident foreign-born workers. Categorized as “essential workers”, they worked throughout the COVID-19 pandemic, while facing high infection risk. Using case-studies in three country contexts – Illinois/USA, the Netherlands, and North Rhine-Westphalia/Germany – we analyzed policy documents, investigative reports, publicly available data, and informal expert consultation to examine structural causes of protection gaps for workers in the meat industry as well as facilitators and barriers to improving occupational safety and health. The Framework Method was applied to systematize and compare the overall data.

Our analysis yields two key findings: First, immigrant workers in the meat industry face similar structural conditions across country contexts, with intersecting immigration- and employment-related precarity, generating gaps in social and health protection and deficiencies in the realization of theoretically held rights. Second, as policy responses to SARS-CoV-2 outbreaks varied, our case-studies showcase fundamentally different approaches to state responsibility for worker wellbeing as part of food supply chain (FSC) governance. The sacrificial-worker approach, observed in Illinois/USA, prioritized industry interests over worker and public health. In the Netherlands, a passive government delegated responsibilities to industry actors who forestalled systemic change through ad hoc adjustments, leaving the core problem of workers’ precarity intact. In Germany, the government leveraged the COVID-19 pandemic as a catalyst for change by enforcing a ban on subcontracting workers in the meat industry, with the potential to fundamentally shift industrial relations and thus address the root causes of worker precarity. Our results highlight economic liberalization and related worker precarity as central determinants of health inequities; and they underscore the imperative for more equitable social and health protection of all workers as part of FSC governance, and as part of food systems transformation for sustainability.

**Supplementary Information:**

The online version contains supplementary material available at 10.1186/s12992-025-01104-9.

## Background

The COVID-19 pandemic has brought into stark relief the inequitable distribution of health risks and protections facing workers around the globe, with immigrants being among the disadvantaged [1, 2]. In the early phase of the pandemic, governments categorized some economic sectors as “essential” and required them to keep operating. The food supply chain (FSC) was considered essential and governments mandated the continuation of agriculture, animal slaughter and meatpacking, food processing, transportation, warehousing, and retail groceries. These sectors were not only required to remain open, but were encouraged to scale up and increase line speeds to meet expected shortages and excess demand [3, 4]. However, the same FSC industries are notorious for inadequately protecting their workers from social and health risks, as part of ongoing economic liberalization and the related precarization of labor [5, 6].

The COVID-19 pandemic has thus highlighted two problems: One problem is the discrepancy between FSC workers’ essential role in our societies, yet a lack of fair treatment of these workers, reflected in the denial of labor rights and equitable social and health protections. Protection gaps, in turn, lead to a second problem: in times of crisis, they compromise not only workers’ safety and health, but also the health of communities and the systemic resilience of food supply chains, local economies and health systems [7].

This study is intended to contribute to the formulation of policy solutions to these problems by identifying facilitators and barriers to policy change toward better protection of worker rights and health. To this end, it examines the social and health protection of immigrant workers in the meat industry in three countries: Illinois/USA, the Netherlands, and North Rhine-Westphalia/Germany; and it analyzes the respective policy responses to SARS-CoV-2 outbreaks in meat plants during the pandemic’s first phase. The similarities and differences between the three country contexts offer an opportunity for comparative research. Across the three sites, the meat industry is an important economic player, sharing similarities in terms of employment structures and working conditions. Also, across the three sites, the meat industry was severely affected by high levels of SARS-CoV-2 infection. Our study highlights differences in the policy responses to SARS-CoV-2 outbreaks. We postulate that the COVID-19 pandemic has elucidated systemic problems at the intersection of immigration, work precarity, and health that apply especially, albeit not uniquely, to the meat industry. Our study uses the meat industry as a pertinent case-study to gain insights from a comparative policy analysis that captures health, immigration, and employment policies as well as governmental responses to SARS-CoV-2 outbreaks in abattoirs; to contribute to an understanding of variations in the rights and protections conferred to precariously employed immigrant workers; and to formulate policy recommendations in line with FSC governance that balances national, societal and worker interests.

The following sections will provide background information on the meat industry in the three country contexts, including characteristics of the labor force, employment structures, occupational safety and health risks, the impacts of the COVID-19 pandemic and the countermeasures taken, as well as the epidemiology of SARS-CoV-2 outbreaks in abattoirs. After a description of our methodology, we present the results of the comparative policy analysis. The final section discusses similarities and differences between the three case-studies by drawing on comparative political economy- and industrial relations-literature, and infer lessons for social policy and FSC governance.

### The meat industry in Germany, the Netherlands and the USA: workforce, employment structures, and occupational health risks

The meat industry has been described as a showcase for the precarization of labor as part of wider processes of global economic liberalization [see, e.g., 8–10]. Precarization has allowed large employers, conglomerates, and retailers to compete on a globalized meat market and meet the growing demand for “just in time” production of meat, worldwide, by externalizing competitive pressures and related risks and costs and/or transferring them down the supply chain [11–14].

Illinois/USA, the Netherlands, and North Rhine-Westphalia/Germany are significant meat producers. Their food markets are characterized by strong competition and oligopolistic tendencies which keep meat prices low while ensuring high profit margins [15–17]. The concentration of the industry in a few conglomerates over the last decades was accompanied by a shift in employment structures from permanent work contracts to indirect and temporary arrangements, including subcontracting and zero-hours work contracts [18, 19]. Defined by employment insecurity, low income and rewards, and a lack of de facto rights and protections, these employment structures can be summarized as precarious [20]. The workers in these precarious employment conditions tend to be foreign-born, brought for temporary staffing from other countries or residents of marginalized communities, as autochthonous workers tend to avoid this work [21–23]. Overall, these developments in the meat industry have been described as a systematic engineering of worker precarity vis-à-vis increasingly powerful employers [3, 6, 8, 12].

Animal slaughter and meatpacking are highly controlled activities, requiring high-intensity, repetitive, ergonomically challenging, physically hazardous, and distasteful work. Hazards in the workplace include the handling of panicking live animals and exposure to high noise levels, dangerous equipment, slippery floors, low temperatures, and high line speeds. Musculoskeletal disorders and injuries are common among the workers. In addition, they are exposed to hazardous chemicals (e.g., ammonia) and biological hazards, including feces and blood [24]. Psychosocial stressors include the need to cope with animal suffering and death, low workplace civility such as humiliating and intimidating treatment by supervisors [25], precarious conditions of employment, and contempt for this type of work [21]. On top of these innate risks, the meat industry is notorious for exploitative conditions and deficits in workers’ rights and occupational safety and hygiene [3, 5, 6]. Working in animal slaughter and meatpacking is thus inherently hazardous and, moreover, influenced by multiple upstream factors that compound occupational risks.

### COVID-19 in the meat industry

Across different country contexts, workers in the meat industry were hard hit during the COVID-19 pandemic, experiencing high morbidity and mortality rates, second only to healthcare workers [26–28]. Furthermore, there is evidence of SARS-CoV-2 spread from meat plants into communities [6, 29]. Previous research had related hazardous living and working conditions in the meat industry to more distal factors such as employment structures and industrial relations [30–32]. However, research on SARS-CoV-2 outbreaks in meat plants has largely focused on the role of working conditions for infection dynamics. It shows that the proximate cause of high infection rates was a combination of indoor, high-density work (workers standing in close proximity to co-workers), exertional tasks (causing heavy breathing, rapid heartrates and an inability to wear a mask during work), air-recirculation, and cold and humid environments [6, 28, 29, 33].

Given that both proximate and distal health risks were long known, SARS-CoV-2 outbreaks in meat plants were foreseeable and preventable. Nevertheless, massive outbreaks occurred in meat plants across different country contexts [34]. The high incidence of contagion and – to the extent known - morbidity and mortality among workers bespeaks a lack of attention to infectious disease principles and occupational health measures in the first phase of the pandemic. This paper considers this failure to protect workers within the context of their immigration- and employment-related precarity; and it compares the policy responses to SARS-CoV-2 outbreaks in the meat industry in three pertinent country contexts, with the goal of contributing to a more informed, effective and just response to future public health crises.

## Methods

We conducted a comparative analysis of social and health policies for immigrant workers in the meat industry in the three country contexts (Illinois/USA, the Netherlands, and North Rhine-Westphalia/Germany), specifically regarding pandemic measures, workers’ rights and social rights. The analysis is further informed by continuous informal consultations with experts and policymakers. It draws on findings from previous research on the impact of COVID-19 measures on migrant workers in the Netherlands and Germany [35, 36]. The overall analysis applied the Framework Method, whose highly structured approach is suitable for joint qualitative analysis in interdisciplinary teams [37]. The research team developed an original framework for comparative policy analysis. The development of the framework capitalized on the interdisciplinary composition of the research team, which brought together the perspectives of occupational health, public health, social law, sociology of work, and migration studies as well as the team members’ familiarity with the different country contexts. The framework captures (a) intra-EU mobility and immigration law, (b) labor law, (c) social and health policies, including occupational safety and health (OSH) regulations, and (d) specific pandemic measures. It served to generate a matrix, with the above categories as rows and the “cases” (country contexts) as columns (see supplementary material, Table 1).

The matrix was applied to each country context by the respective researchers to systematize data collection and presentation. The following sources and types of information were searched: (1) policy documents (e.g., laws, executive orders, administrative guidelines, protocols of parliamentary hearings and relevant statistics–were retrieved from the institutional websites of governments, Departments of Labor, Departments of Agriculture, Public Health Agencies, Occupational Safety and Health Agencies, and parliaments); (2) investigative reports from news outlets and NGOs; and (3) websites of public interest and private organizations (business associations, labor unions), and agencies dealing with or collecting information on workers’ claims (e.g., FairWork Foundation, Workers’ Compensation Commission, Faire Mobilität of the Federation of German Trade Unions), which provided raw data and descriptions of policies and practices.

The above data were complemented and triangulated with information obtained through informal consultations with key informants in each setting. These included persons in government (e.g., departments of health, labor, and immigration), and representatives of social security agencies, non-profits, and labor organizations. The Dutch case-study additionally drew on insights from a previous qualitative study on EU mobile worker’s rights in the Dutch meat industry during the COVID-19 pandemic [19]; and the German case-study was informed by prior research on the conditions of EU mobile workers in the meat industry and agriculture during the COVID-19 pandemic [36].

Data were entered into the matrix, summarized by category [37]. Throughout data collection and analysis, experiences with applying the analytical framework as well as ongoing policy developments were discussed among the research team via videoconferencing, and the framework was revised and refined accordingly [37]. This paper reflects the final amalgamation of our joint work.

## Results

Our analysis highlights that, while the structural conditions of immigrant workers in the meat industry seem broadly similar across our three country contexts, the policy responses to SARS-CoV-2 outbreaks vary markedly, with each country context showcasing a fundamentally different approach to worker protection and the related role of government.

### Illinois, USA: the sacrificial worker approach

In the USA generally and in Illinois specifically, inequalities in COVID-19-related health risks reflect long prevalent social inequalities affecting immigrant, African American, Hispanic, and rural workers, who are over-represented in the meat industry. Resettlement programs channel refugees into frontline work, including in meat plants [23, 38]. Over one third (38%) of the workforce in the meat industry is foreign-born; 35% identify as Hispanic and 22% as Black, compared to 19% Hispanic and 13% African American across the USA. Half of the workers are on temporary contracts or work informally [39]. Meat companies often employ workers at low wages and with minimal social benefits. Almost half (45%) of the workers live in low-income families and around 12% live below the poverty line; these levels are twice as high as in other workers [40]. There is no guarantee of job security and workers can be laid off without notice. Down from 90% in 1952, only 18% of meat-processing workers belong to unions [24].

During the first phase of the pandemic alone, five large US American meat companies saw approximately 60,000 confirmed SARS-CoV-2 infections and 298 deaths among their workforces [41, 42]. Up to 310,000 additional COVID-19 cases and 5,200 deaths have been attributed to communities’ proximity to meat plants, equivalent to 6–8% of all cases and 3–4% of all deaths in the USA at the time [6, 43].

Throughout the COVID-19 pandemic, there was a lack of attention to the well-known hygiene hierarchy of preventive/protective measures that prefers engineering controls (e.g., improving ventilation, slowing down lines) and administrative controls (e.g., thinning the census of workers on a given shift, worker screening) above use of personal protective equipment [44]. Inadequate screening and testing protocols and the lack of provision of masks further increased the workers’ risk of infection. Employment conditions were also at fault: the lack of paid sick leave, job insecurity, and penalizing workers for missing shifts induced workers to come to work, even when sick. Limited healthcare access — due to lack of insurance, unfamiliarity with the local health system, and distance to healthcare providers — further contributed to spreading the virus among workers and into communities. Moreover, many members of this workforce live in crowded housing and share transportation to work, thwarting basic public health advice to isolate and quarantine.

Workers’ compensation (insurance and benefits in case of a work accident) varies across US states. In Illinois, workers who have been employed by one employer for longer than 14 weeks are eligible for workers’ compensation insurance. Illinois issued a “rebuttable presumption” of work-relatedness for frontline/essential workers, including FSC workers [39], which theoretically makes it harder for employers to refuse compensation. Claims are wending their way through the arbitration system and it is not yet known how many claims in the meat industry have been compensated, to date.

On a local level, the inadequate pandemic preparedness and response in workplaces can be attributed to the fact that local health departments were tasked with these measures. Not only do they have limited expertise in workplace investigations, but they often avoid confronting business owners, who may play an important role in the local economy. Moreover, local health departments operate independently of the Occupational Safety and Health Administration (OSHA) under the US Department of Labor, which is responsible for setting workplace standards and enforcing them in the meat-processing industry. The transfer of responsibility to local health departments hampered data sharing and systematic health monitoring. Several additional factors prevented effective health monitoring: generally, occupational health surveillance is not integrated with infectious disease surveillance; neither local nor state health departments nor the federal Center for Disease Control and Prevention collected systematized or standardized information on the workplace as a site of exposure. This prevented identification and a generalized response to the most hazardous sectors. Public Health’s data on COVID-19 in specific industries such as meat-processing thus needed to be patched together from an array of sources, and likely undercounted these cases. In fact, the only accessible count of cases in the food supply chain in Illinois was produced by a news outlet [42].

On a national level, effective public health efforts were upset by the federal authorities, thus allowing COVID-19 to spread through the population. Notably, while general health and safety standards were compulsory during the pandemic, the US Supreme Court prohibited OSHA from promulgating an Emergency Temporary Standard for COVID-19 aimed at reducing infection, even in healthcare occupations. This reduced OSHA’s authority to more effectively control the spread of SARS-CoV-2. In fact, meat plants were allowed to increase their line speeds - which has been shown to be linearly related to injury - to increase meat production [4]. OSHA did respond to complaints and outbreaks, using the “General Duty Clause” to fine non-compliant employers. However, by September 2020, the fines totaled a mere $29,000 for 1500 infections and 12 deaths [39].

In May 2022, Congressional hearings uncovered evidence of influence peddling on the part of the major meat-processing companies. At the time, the head of the US Department of Agriculture was the owner of one of the largest meat companies in the USA. While massive outbreaks were ongoing and evidence of SARS-CoV-2 spread from meat plants into rural communities accumulated, the companies sought insulation from liability for worker deaths. The US president issued an Executive Order to prevent state and local health departments from regulating meat companies under the “Defense Production Act”, and once more prohibited OSHA from applying an emergency temporary standard for any sector of the economy. Overall, the Illinois case-study showcases a sacrificial worker-approach, where the federal government prioritizes industry interests over worker wellbeing during a public health crisis, at the price of the workers’ and the public’s health. Negative attitudes toward immigrants, in general, were promoted by the highest levels of government during the period of the pandemic and, likely, also played a role [45]. The fall of unionization in meat-processing lowered support for workers, as did the industry’s move to rural areas where companies exert outsized political power as the major economic drivers [46].

### The Netherlands: inaction through protraction

In EU member states such as the Netherlands and Germany, the expansion of the European Union (EU) in 2004 and the EU-wide free movement of workers and services led to the supply of labor from East- and South-East-European member states such as Poland, Romania, and Bulgaria [8, 12, 13]. The Dutch meat industry is highly dependent on EU mobile workers, who account for 90% of workers on the production floor. EU mobile workers reside and work legally in the Netherlands and, as EU citizens, are entitled to equal social rights. Yet, their precarious employment and dependency on the employer hinder equitable social and health protection. The COVID-19 pandemic and related measures exacerbated these conditions.

Most EU mobile workers in the Dutch meat industry are hired via temporary employment agencies located in the workers’ home country, the country of employment, or another EU member state. The meat plants, in which the workers are put to work, are thus not formal employers but contractors of the respective temporary employment agency. As such, they have no direct contractual relationship with the workers and are largely exempt from employer obligations. In 2020, temporary contracts made up half of all work contracts and up to 100% in some meat plants [19]. The workers are theoretically covered by the collective labor agreement applicable to the entire meat sector. Yet, their zero-hours work contracts stipulate payment and social benefits only for the number of hours worked; and they allow for dismissal should no work be available or if the worker calls in sick. Especially in the first phase of temporary agency contracts, working hours are not guaranteed and contracts can be dissolved easily, leading to loss of employment and, frequently, to a loss of employer-organized housing and healthcare. This results in employment and income insecurity and in encompassing dependency of the workers on their employers. The Dutch state, in turn, has only limited oversight via sporadic inspection [47].

To add to the fragmentation and complexity, EU mobile workers are not registered with a Dutch address, but in a special register created for ‘non-residents’. Some EU mobile workers are accommodated in neighboring Germany for cheaper rent and often in desolate conditions whilst working in meat plants on the Dutch side of the border. While such cross-border work and housing arrangements are well established in the region, cross-border enforcement of housing and public health standards is nascent [36, 48, 49]. During the COVID-19 pandemic, this multi-fragmented system had important public health consequences. The Dutch authorities had no information on how many EU mobile workers were employed in Dutch meat plants, where they lived, or how to reach them. Rather than addressing this lack of oversight, the Dutch public health authorities delegated responsibilities for health information and pandemic measures to employers and contractors, while ordering labor inspectors to stay home for their own safety. Employers and contractors were thus in charge of organizing SARS-CoV-2 testing. Interview data suggest flaws in the implementation of pandemic measures and breaches of workers’ rights, reflecting conflicting economic and public health interests. For instance, workers reported testing after a shift, rather than before starting it; some were asked to return to work from quarantine earlier than recommended. Others had to take vacation leave instead of being paid quarantine or sick leave. The Dutch Labor Inspectorate confirmed that, when meat plants were temporarily closed due to SARS-CoV-2 outbreaks, directly employed workers were paid compensation, whereas temporary workers were not paid.

The workers themselves were hesitant to test or report illness because of the risk of losing income or their job. Some continued to work despite being sick. Many stated that they were unable to adhere to safety and health instructions on the work floor, during transportation, or in their employer-arranged housing. Yet, they were afraid to complain and, on top, it was often unclear who to complain to, given the unclear lines of responsibility among employers and contractors. The authorities’ reliance on industry actors for the voluntary implementation of pandemic measures allowed them to evade their responsibilities while reinforcing the workers’ dependency [50].

German authorities voiced their concerns that Dutch temporary staffing agencies were taking advantage of the supervisory vacuum in cross-border labor arrangements and thus facilitating the uncontrolled spread of SARS-CoV-2 [51]. In this context, two outbreaks in Dutch meat plants triggered a swift change of the Dutch Public Health Act, empowering the Heads of the Region to close businesses and impose quarantine [52]. After the temporary closure of the said two meat plants, however, the Heads of Region used these powers sparingly. Instead, the largest Dutch meat companies self-committed to more direct hires and the improvement of workers’ conditions. This concession was accompanied by further adjustments, including a two-months statutory minimum income guarantee for newly arriving EU mobile workers, and a four-week transition period to vacate employer-organized accommodation upon termination of the work contract [53].

Notably, apart from the change of the Dutch Public Health Act, most measures taken in the Dutch context present ad hoc adjustments and concessions by non-state actors, while the central government remains largely passive, protracting long-envisaged and rather minor reforms (such as legal changes which would separate workers’ rental and work contracts, tighten temporary employment agency regulation, and improve precarious workers’ access to labor courts). The Dutch case thus illustrates how the industry’s “patching up” of a multi-fragmented system may alleviate its gravest symptoms; yet, ultimately, it allows for continued governmental inaction and thus leaves the systemic problem of EU mobile workers’ precarity intact.

### North Rhine-Westphalia, Germany: COVID-19 as a catalyst for systemic change

As in the Dutch case, the German meat industry relies heavily on subcontracted workers from East and South-East European countries. Temporary-staffed workers have been providing about half of the manual labor in German meat plants; in some plants, they comprised up to 100% of the workforce [54]. With subcontracted workers not being included in meat industry employment statistics, no comprehensive information on the number and profile of EU mobile workers in the German meat industry is available [18].

Until 2014, the German meat industry used EU mobile workers via posting; that is, the workers were employed by temporary employment agencies in other EU member states and put to work in German meat plants at conditions and pay below German minimum standards. In case of need, workers often found themselves without any social security, as contractors refuted liability and subcontractors were difficult to identify amidst complex multilayered subcontracting arrangements. For instance, workers who returned to their home country following an accident with consequent incapacity to work were often unable to claim outstanding wages and compensation [55]. Years of negative media coverage and related political pressures – including a formal complaint of unfair competition by the Belgian government in 2013 [31, 54] - resulted in the government’s determination of a statutory minimum wage in 2015 and the 2017 expansion of contractors’ liability for accurate payment of wages and social insurance contributions. Anticipating that these legal changes would render posting unattractive, the German meat industry required temporary employment agencies to relocate to Germany, included subcontracted workers in the applicable collective labor agreement, and set a branch-specific minimum wage. These actions were accompanied by an extensive image campaign, as part of which 52 German meat companies pledged to increase the proportion of direct hires among their workforce [54, 55].

Yet, reality saw little change, neither in the proportion of subcontracted workers nor in their conditions. Rather, unions collected evidence on pervasive violations of legal requirements, and the authorities’ inspections similarly indicated “institutionalised non-compliance on employment conditions” [54, p. 10], most commonly in the form of illegal wage deductions, breaches of work time regulations, occupational safety and health shortcomings, and inadequate housing [3, 54–56]. In some cases of legal disputes, expert evaluators were not admitted to worksites as meat companies claimed that conflicts between contractors and their employees were not their concern and they were therefore under no obligation to admit third parties to their premises [55]. These findings underscore that subcontracting continued to facilitate abusive practices, allowing employers and client firms to apply legal loopholes and circumvent standards by exploiting employment- and migration-related power imbalances (such as employment arrangements that bind workers to single employers, language barriers, lack of familiarity about how to exercise their rights, and lack of local social support) [54, 57]. By way of comparison, few deficits were found in meat plants with directly hired employees [54].

Then, in the early phase of the COVID-19 pandemic, the implementation of pandemic control measures affected EU mobile workers in German meat plants in particular and unique ways, aggravating their socio-economic, living and working conditions. As some meat plants were temporary closed in response to SARS-CoV-2 outbreaks, workloads were shifted to those plants that remained open, leading to greater time pressure and longer working hours, at the expense of worker safety and health. In some contexts, to avoid closures and lockdowns, workers were put in “labor quarantine”: whilst upholding production in the meat-processing plant, EU mobile workers were allowed only in their living quarters, their workplace or commuting between the two; but they were banned from the public space, including supermarkets, parks and public transport, while no provisions were made for supplying food and basic necessities [36]. These measures reproduced a public discourse focused on racialized stigmatization and “othering” rather than the structural conditions that gave rise to SARS-CoV-2 infection and spread [3, 36].

The discourse changed as further SARS-CoV-2 outbreaks in meat plants impacted the surrounding communities. One large outbreak in an abattoir in North Rhine-Westphalia, for example, compromised the food supply and local healthcare system and eventually led to a local lockdown for over 600,000 local residents [18, 58]. Public indignation rose as the respective meat company demanded state compensation for economic losses, while its haphazard handling of preventive measures came to light. Workers reported, for example, that no protective gear was provided and that the conditions on site and during transport were only improved during inspections and then returned to “normal” [36]. This shifted the public debate back to the meat industry’s exploitative practices and evasion of liability.

The federal government seized this momentum in May 2020 to declare its determination to resolve the problem of working conditions in the meat sector. By summer, it had submitted draft legislation to the German parliament, expressing the need to establish “clear lines of responsibility” in order to reliably protect workers’ rights [59, p. 25]. In December 2020, the German Minister of Labor declared in front of the German Parliament: “We are thoroughly cleaning up the meat industry because the human dignity of employees is at stake” [60]; and one week later the parliament passed the federal Occupational Health and Safety Control Act (Arbeitsschutzkontrollgesetz). This federal law restricts the subcontracting of workers in the industry’s core areas of slaughtering, cutting, and meat-processing. It also stipulates the electronic recording of working hours, and increases the frequency of inspections and penalties for employer non-compliance. It sets standards for workplaces and housing for all economic sectors, beyond the meat industry, and clarifies the employer’s respective responsibilities. As of 2021, meat companies in Germany must employ their workers directly and accept all related responsibilities, including social and health insurance coverage, OSH and pandemic measures, and adequate accommodation for non-local workers. The states of North Rhine-Westphalia and Lower Saxony formulated an additional 10-point-plan, which lists concrete steps for “systemic change” in the meat industry [61]. Alongside the aspect of fair competition, government representatives emphasized worker rights as the main motive of the reforms, declaring, inter alia, that “some meat companies had, for too long, operated in a system of organized irresponsibility” and that “the excess COVID-19 cases among the workforce of abattoirs… are evidence that systemic change in this industry is overdue” [62]. At the same time, media analyses show that the “othering” of East European immigrant workers remains one thread of the public discourse that can be mobilized for populist purposes in times of crises [36].

In June 2021, the government of North Rhine-Westphalia passed the Housing Strengthening Act (Wohnraumstärkungsgesetz) at the state level. The law sets housing standards, specifies employer’s responsibilities, and expands municipalities’ capacities to intervene in case of problematic accommodations also in case of cross-border arrangements [63]. The three neighboring states Germany, the Netherlands, and Belgium further strengthened cross-border cooperation and enforcement by establishing a designated taskforce. One of the results of the measures taken to control the COVID-19 pandemic was, consequently, the establishment of a cross-border network between authorities for the control of accommodations on the German side of the border as well as of businesses on the Dutch or Belgian side. This highly complex and time-consuming procedure has led to the prosecution of some of the most flagrant cases of migrant labor exploitation in recent years. Exchange of information and data was facilitated through the institution of a liaison office in the Netherlands, which examines the relevance of information to national law or emergency preparedness and, if necessary, passes it on to the competent authority. This way, cross-border regulatory violations can now be prosecuted in conjunction [36].

The new regulations have been assessed to be largely successful by trade union organizations and other organizations providing social counseling to mobile workers [36, 55]. Previously subcontracted workers have been directly hired by the same companies. There is evidence for more accurate working hours and pay. Increased inspections and higher fines for violations appear to have contributed to improvements, as follow-up inspections found markedly fewer deficits in the controlled facilities [36, 64]. Some challenges remain, such as entrenched hierarchies among workers, supervisors and human resource managers (many of whom previously operated temporary employment agencies, “handling” the same workers for the same meat plant) and low workplace civility [36, 54, 55, 65]. But overall, some authors note, the ban on subcontracting has fundamentally changed industrial relations in the German meat industry, as evidenced by the newly hired workers’ instant organizing for pay raises. Joint action with the trade unions asserted a national and industry-wide binding minimum wage through collective bargaining and strikes in 2020 [55]; and in 2021 an agreement on sector-wide minimum working standards, whose legal enforceability is yet to be confirmed by the federal government [66]. The German case-study thus showcases how the COVID-19 pandemic served as a catalyst for systemic change that addresses precarious employment arrangements as the root cause for insufficient social and health protection for immigrant workers.

## Discussion

This paper aims to contribute to a better understanding of health risks and protection gaps for immigrant workers in FSC industries. Using the meat industry and three different country contexts as case-studies, it compares divergent policy responses to the COVID-19 pandemic to elucidate facilitators and barriers to improving worker protection. In doing so, our study yields two main insights: First, it illustrates how intersecting migration- and work-related precarity generates deficits in immigrant workers’ social and health protection. Second, it identifies fundamental differences in governments’ acceptance of responsibility for worker wellbeing as part of FSC governance. These differences may help understand the structures and dynamics that can create a “virtuous circle” [67, p. 13] to engage stakeholders in creating and maintaining decent work conditions. To this end, the following sections will discuss the above two insights in light of the existing political economy and institutionalist literature.

The association between precarious employment and health inequities has been extensively documented and comprehensive conceptual frameworks developed [see, e.g., 68–70]. However, despite previous calls to consider migration in this body of research [71] and to consider work as a central determinant of immigrant health [72], migration often remains poorly integrated in conceptual frameworks and relevant research is fragmented across disciplinary silos [73]. Our study contributes to a nascent body of research on the nexus of all three arenas – migration, work, and health [see, e.g., 74–77) – by exemplifying how migration-related instances of marginalization are part of the precarization of labor in the meat industry, and how this played out during a public health crisis.

A first insight from our case-studies is that employment precarity is the key factor that increased workers’ exposure and risk of infection with SARS-CoV-2. They illustrate how precarious employment prevents workers from asserting legally held rights, inter alia by allowing employers and staffing contractors to “pass the buck” to one another concerning their responsibilities vis-à-vis the workers. This can lead to gaps in preventive measures and social protections when neither employers nor contractors provide protective equipment, implement workplace-based OSH training or interventions, or pay into social and health insurance [19]. It also fueled the spread of infection into communities [6, 43]. Migration status and the related power differentials amplify employment precarity – for instance, if immigrant workers’ access to support mechanisms is hindered by lack of familiarity, trust and language barriers [78] – and contribute to poor occupational health outcomes mainly through this interaction [2, 79].

To be exact, our analysis points to the role of legal statuses in enabling or hindering workers’ mobilization of rights. In the case of Illinois/ USA, many workers in the meat industry lack immigration and work authorization status and thus have no power to assert rights. By way of comparison, the status of EU mobile workers in abattoirs in Germany and the Netherlands is more secure and their conditions are extensively regulated. This should in theory translate into better protection of rights. Still, EU mobile workers tend to fall between the cracks of EU, national and local jurisdictions, which create loopholes in terms of liability, legal protection, and enforcement [80]. In the short run, these loopholes allowed industry actors to create “zones of exception” from local employment rights and regulations. In the long run, they undermined the concept of labor rights altogether by setting in motion a gradual reinterpretation of institutional rules that allows for differential rights and protections [14, p. 14]. Hence, while one might expect EU citizenship to provide equitable rights and protections within the EU, our study illustrates how, on the contrary, EU mobile workers remain marginalized and positioned as “outsiders” [12], for other stakeholders’ economic benefit.

With regard to future research, our study points to the importance of a nuanced consideration of migration as a multifaceted phenomenon; and it highlights the importance of intersectionality as a theoretical framework that helps overcome “methodological nationalism” [81] by considering the complex interrelations among migration status(es) and other determinants of work precarity and occupational health inequities, while remaining oriented toward power structures. Such a theoretical lens will be particularly important to avoid the pitfall of “migrantizing” the problem of precarious work, and instead emphasize common causes related to worker justice across different social categories. With regard to action for workers’ rights, our study strengthens calls for broadening solidarity and social movements, across narrow categories defined by citizenship, legal status or social class, “as the condition of precariousness is increasingly one shared to greater or lesser degrees by workers generally.” (67, p. 2, see also 82) With regard to law and policy, our analysis indicates that EU citizenship is not yet geared to transient stays and continued reforms will be necessary to realize equitable social citizenship within the EU.

This leads us to our study’s second main insight, which relates to the role of governments in addressing essential workers’ safety and health during a public health emergency. A large body of literature examines the expansion of employment precarity worldwide and the role of institutions, therein [14, 67, 83, 84]. The COVID-19 pandemic has demonstrated the social and health risks of the precarization of labor for workers and communities. Our analysis highlights that when these risks became clear, government responses diverged markedly: from sacrificing workers, to inaction, to systemic change toward better protection. In line with the above literature, these divergent policy responses manifest fundamental differences “with respect to the role of the state versus the social partners with regard to… regulation and control” (83, p. 535, see also 13, 84); or, more concretely, with respect to governments’ approaches to FSC governance and to the assumption of responsibility for worker rights and wellbeing as an inherent part, thereof.

In the case of Illinois/USA, the federal government’s siding with industry reflects an extractive capitalist approach to FSC governance that pursues profit maximization as its primary goal, while actively sidelining worker rights. It is not lost on the workers themselves that they are “not being valued as people but as tools to produce a product” [85, p. 1555]. Previous studies note that, in the USA, due to weak regulation of product and labor markets, employers “did not face great constraints if they opted for ‘low road’ business strategies” [84, p. 337]; that is, for cutting costs by downgrading employment and workplace conditions. In our study, rather than being merely permissive, the US federal government appears to be actively promoting such strategies. During a public health emergency such as the COVID-19 pandemic, such an approach to governance of the FSC, and, likely, other economic sectors, translates into sacrificing workers’ and public health to industry interests.

By comparison, our analysis characterizes the Dutch government’s approach to FSC governance as inaction through protraction. The Dutch government has remained passive in the face of a multi-fragmented system of migration, labor, and housing arrangements that has stripped it of oversight and control, in the meat industry as well as in other sectors such as construction [86]. With minor legislative changes “stuck in the pipeline”, the Dutch government reinforces its passive role during the COVID-19 pandemic by delegating further responsibilities to employers and contractors, thus amplifying worker precarity. With the accumulation of SARS-CoV-2 outbreaks – and potentially with an eye to developments unfolding on the German side of the border – the Dutch meat industry preempts public backlash and appeases the government by “patching itself up”. Forestalling more profound policy change, this approach leaves the systemic problem of immigrant worker precarity intact. By the same token, it has been suggested, such “band-aid solutions” [87] fail to ensure the provision of labor for a resilient FSC in the long run, and especially in case of future public health crises.

The German case offers somewhat of a silver lining. Here, federal and state governments used the COVID-19 pandemic as momentum to re-regulate employment, workplace, and housing conditions with industry-specific legislation. Importantly, this policy change was long in the making. In line with previous research [3, 18, 32, 54], our study traces a prolonged “arms race”, in which various attempts to improve workers’ conditions are met by industry actors’ determination and creativity to maintain the status quo in practice, not least of which earns Germany a poor image among the EU member states as regards FSC governance [8, 9, 31, 54]. Eventually, the COVID-19 pandemic provided the government with a window of opportunity to enforce a fundamental transformation of industrial and power relations [88]. Its justification of this intervention reflects an approach to FSC governance that considers workers’ rights and wellbeing alongside economic interests and political interdependencies within the EU common market. It will be important to study the impact of these new regulations.

Ultimately, our case-studies thus lend support to the claim that country-specific industrial relation institutions shape differences in workers’ conditions generally [13, 84], and differential impacts of the COVID-19 pandemic specifically [89]. Dobbins et al., for example, argue that “industrial relations institutions matter for shaping policy choices across different countries…[Therefore, ] the precise implications of the COVID-19 crisis for workers and work outcomes are embedded in country-specific institutional variations within capitalism as a global economic system” [89, p. 115]. From this perspective, our findings substantiate previous research, which showed that coordinated market economies like Germany and the Netherlands protect their workers better than liberal market economies like the USA [82, 84].

Such national-level differentiation leaves further questions open: For instance, why did the COVID-19 pandemic serve as a window of opportunity for systemic change in the specific case of the German meat sector, but not in other industry contexts in Germany? What can explain within-country variations and changes in industrial relations institutions? Here, the existing literature offers more granular-level explanations, which may also provide insights into “what works” for asserting worker rights against economic interests.

Some authors highlight that, just as much as industrial relation institutions shape the different stakeholders’ actions, the actions and strategies of local players constantly shape and re-shape industrial relation institutions; inter alia, by using, subverting, challenging, circumventing, or non-complying with rules [14, 67]. From this perspective, our case-studies trace employers’ continuous efforts to de-regulate; for example, by circumventing regulations (the “arms race” in the German case) or forestalling re-regulation through appeasement (as in the Dutch case). Conversely, the German case also offers an example of how various players leverage “multiple forms of power and agency” (67, p. 118, see also 89) toward an institutional change that was evaluated as a “landslide” in industrial relations in the German meat industry [88]. The changing role of unions herein may be particularly instructive, as they move from a lack of inclusion and solidarity with mobile workers (which, according to Wagner & Refslund, facilitated the deterioration of workers’ conditions in the German meat industry in first place) toward a multi-pronged approach to mobilizing for immigrant workers’ rights, including the operation of dedicated departments, linguistically diverse teams, and media and social media campaigns [13]. This may serve as another example of how successful worker movements today “are based on building bridges between identity-based social movements and labour movements” [67, p. 6], thus bringing together a broader basis and more diverse forms of power resources and collective action. Given the scarcity in knowledge about effective structural level interventions to sustainably improve immigrant workers conditions [79], future research ought to dig deeper into such success stories and good practices to better understand policy change, and to leverage that understanding for social impact.

Our study has several limitations. As with many comparative studies, differences in the data collection between the three research sites, related to different data sources, availability and accessibility, were unavoidable. However, we do not think that these differences have led to any systematic bias. Moreover, authors in all three country teams possess long-term practical knowledge of the subject matter, as they are involved in relevant expert consultations and policymaking processes with governments and international organizations on a continuous basis. This allowed them to critically assess the quality and trustworthiness of all collected data, further reducing the risk for bias and benefiting the overall study. Policies on immigration, work, and public health are subject to constant change; our analysis cannot but capture and reflect on these developments from the perspective of a certain point in time. Importantly, our data allows for a mapping of policies and policy variations in the three country contexts. Our study did not aim to empirically measure differences in policy outcomes (e.g., infection rates) or explain policy and institutional variations based on empirical findings. While we do offer some explanations for the identified policy differences by relating our findings to the existing literature, further empirical research is warranted to verify these inferences. We furthermore acknowledge that our position as educated, white researchers with little to no experience in precarious manual labor influences our analysis and interpretation of results.

## Conclusions & recommendations

Our study highlights intersecting immigration- and employment-related precarity as a structural cause of deficits in the health and social protection of immigrant workers in the meat industry across country contexts. At the same time, its findings suggest fundamental differences in FSC governance between country contexts, which have, in turn, contributed to divergent responses to the COVID-19 pandemic, with implications for worker and public health as well as systemic resilience. Our study underscores the relevance of a “global power perspective” [81, p. 23] with economic liberalization, worker precarity, and institutional responses as central determinants of health inequities. It calls attention to the imperative for more equitable social and health protection of all workers as part of FSC governance, and of food systems transformation for sustainability. Our results bring forward five key recommendations.

First, comprehensive and sustainable improvements of immigrant workers’ social and health protection in the relevant FSC industries will require tackling systemic problems like unequal power relations, corporate social irresponsibility, and cronyism [90, 91]. In this regard, our comparative analysis pinpoints the key role of FSC governance that considers workers’ wellbeing and rights alongside economic and political interests. Industrial relations institutions matter, to the extent they open up to include immigrant and minority workers. Governments have an important role to play by enacting binding legal frameworks that empower workers to assert their rights and clearly establish the corresponding employer duties and duty holders.

Second, better legal frameworks need to be accompanied by strong proactive enforcement of workers’ rights, while at the same time firewalling data exchange between OSH and immigration agencies. Generally, there is an urgent need to improve the availability and quality of data at the intersection of occupational, public and migration health, while ensuring adequate data protection; inter alia by integrating occupational health surveillance, infectious disease surveillance, and public health surveillance and by linking the respective data. Data collection should be more informed by existing knowledge on health inequalities (e.g., collect information on relevant social categories such as migration and employment status); and it should collect data in ways that facilitate comparative and intersectional analyses.

Third, given the transnational character of today’s FSC industries and their labor arrangements, legal frameworks must be designed and effectively function as transnational social protection schemes (35). Other mechanisms to strengthen workers, such as unionization, similarly need to be adapted to a transnational market, for instance, by offering labor unions standing in cross-border settings.

Finally, our study underscores the importance of ongoing debates on food systems transformation for greater sustainability and social justice. In these debates, to date, sustainability and social justice are addressed in terms of environmental implications, consumer protection, the distribution of resources and produce, and occasionally animal welfare [92–94], while workers’ rights are absent. Critical grassroots organizations like the People’s Health Movement have been calling for greater consideration of commercial determinants such as trade liberalization and the associated “evaporation of decent employment” [95, p. 4] as fundamental drivers of health inequity, worldwide. It is time for this call to be picked up by policymakers and the public health community – particularly after the COVID-19 pandemic has put worker wellbeing on the table. This is important not only to protect the public’s health and as a matter of social justice, but also as an essential ingredient of resilient food systems and a necessary precondition for food security for all.

## Electronic supplementary material

Below is the link to the electronic supplementary material.

Supplementary Material 1

## Data Availability

No datasets were generated or analysed during the current study.
