# Supplementary material for: Immigrant workers in the meat industry during COVID-19: comparing governmental protection in Germany, the Netherlands, and the USA
Source: Global Health. 2025 Mar 22;21:10. doi: 10.1186/s12992-025-01104-9 (PMC11929214; doi:10.1186/s12992-025-01104-9)
Supplement: Supplementary file 1 — Supplementary Material 1 [file 12992_2025_1104_MOESM1_ESM.docx]

Supplementary material

Table 1) Matrix for data collection for a comparative analysis of policies concerning labor rights, social security, and occupational safety and health for workers in the meat industry in Illinois/USA, the Netherlands, and North-Rhine Westphalia/Germany

|  | **Illinois, USA** | **Netherlands** | **North-Rhine Westphalia, Germany** |
| --- | --- | --- | --- |
| **General** |  |  |  |
| Definitions and description of work precarity or irregularity (e.g. temp/ posted/ subcontracted/ unauthorized work) |  |  |  |
| Living conditions (housing, transportation, etc.) |  |  |  |
| **Immigration and labor** |  |  |  |
| Who are the workers? (e.g. migration status, ethnicity, gender) |  |  |  |
| What are the job tasks of precariously employed workers? |  |  |  |
| **Social benefits coverage** |  |  |  |
| Do precariously employed workers have access to… |  |  |  |
| health insurance? |  |  |  |
| paid sick leave? |  |  |  |
| worker compensation? |  |  |  |
| disability benefits? |  |  |  |
| pregnancy/childbirth benefits? |  |  |  |
| **Occupational safety and health** |  |  |  |
| Do in-house occupational services have a mandate for precariously employed workers? |  |  |  |
| **During COVID-19 pandemic** |  |  |  |
| What pandemic measures have been taken in meat plants? |  |  |  |
| Who has/takes responsibility for precariously employed worker's safety and health? |  |  |  |
| for providing protective gear? |  |  |  |
| for testing? |  |  |  |
| for vaccinations? |  |  |  |
| Is paid sick leave available? |  |  |  |
| for illness? |  |  |  |
| for quarantine? |  |  |  |
| Is there worker compensation for COVID-19? |  |  |  |
| Does it cover COVID-19 at all? |  |  |  |
| Does it cover hospitalization? |  |  |  |
| Does it cover rehab for long covid? |  |  |  |
| Does it cover time lost from work? |  |  |  |
| Did healthcare coverage change? |  |  |  |
| How did OSHA/enforcement deal with COVID-19 outbreaks in meat plants? |  |  |  |
| Are there links/exchange of information between different sectors and authorities (e.g., OSHA and immigration authorities, cross-border exchange of information)? |  |  |  |
